# L1077P CFTR pathogenic variant function rescue by Elexacaftor–Tezacaftor–Ivacaftor in cystic fibrosis patient-derived air–liquid interface (ALI) cultures and organoids: in vitro guided personalized therapy of non-F508del patients

**DOI:** 10.1186/s12931-023-02516-0

**Published:** 2023-09-06

**Authors:** Stefania Lo Cicero, Germana Castelli, Giovanna Blaconà, Sabina Maria Bruno, Giovanni Sette, Riccardo Pigliucci, Valeria Rachela Villella, Speranza Esposito, Immacolata Zollo, Francesca Spadaro, Ruggero De Maria, Mauro Biffoni, Giuseppe Cimino, Felice Amato, Marco Lucarelli, Adriana Eramo

**Affiliations:** 1 Department of Oncology and Molecular Medicine, Istituto Superiore di Sanità, Rome, Italy; 2 Department of Experimental Medicine, Sapienza University of Rome, Rome, Italy; 3 Department of Molecular Medicine and Medical Biotechnologies, University of Naples Federico II, Naples, Italy; 4 CEINGE-Biotecnologie Avanzate S.c.a.r.l, Naples, Italy; 5 Confocal Microscopy Unit, Core Facilities, Istituto Superiore di Sanità, Rome, Italy; 6 Dipartimento di Medicina e Chirurgia Traslazionale, Università Cattolica del Sacro Cuore, Rome, Italy; 7 Fondazione Policlinico Universitario ‘A. Gemelli’-IRCCS, Rome, Italy; 8 Cystic Fibrosis Reference Center of Lazio Region, AOU Policlinico Umberto I, Rome, Italy; 9 Pasteur Institute, Cenci Bolognetti Foundation, Sapienza University of Rome, Rome, Italy

**Keywords:** Cystic fibrosis, CFTR, Reprogrammed nasal cells, Nasal organoids, ALI culture, Trikafta, Rare mutation, Theratyping, Personalized medicine, Patient-derived models

## Abstract

Cystic fibrosis (CF) is caused by defects of the cystic fibrosis transmembrane conductance regulator (CFTR) gene. CFTR-modulating drugs may overcome specific defects, such as the case of Trikafta, which is a clinically approved triple combination of Elexacaftor, Tezacaftor and Ivacaftor (ETI) that exhibited a strong ability to rescue the function of the most frequent F508del pathogenic variant even in genotypes with the mutated allele in single copy. Nevertheless, most rare genotypes lacking the F508del allele are still not eligible for targeted therapies. Via the innovative approach of using nasal conditionally reprogrammed cell (CRC) cell-based models that mimic patient disease in vitro*,* which are obtainable from each patient due to the 100% efficiency of the cell culture establishment, we theratyped orphan CFTR mutation L1077P. Protein studies, Forskolin-induced organoid swelling, and Ussing chamber assays congruently proved the L1077P variant function rescue by ETI. Notably, this rescue takes place even in the context of a single-copy L1077P allele, which appears to enhance its expression. Thus, the possibility of single-allele treatment also arises for rare genotypes, with an allele-specific modulation as part of the mechanism. Of note, besides providing indication of drug efficacy with respect to specific CFTR pathogenic variants or genotypes, this approach allows the evaluation of the response of single-patient cells within their genetic background. In this view, our studies support in vitro guided personalized CF therapies also for rare patients who are nearly excluded from clinical trials.

**Supplementary Information:**

The online version contains supplementary material available at 10.1186/s12931-023-02516-0.

## Introduction

Cystic fibrosis (CF) is the most frequent and severe rare genetic disease in Caucasian population, with a recessive modality of inheritance and an incidence observed in 1/2000–3000 newborns [1–3].

More than 2000 variants of the Cystic Fibrosis Transmembrane Conductance Regulator (CFTR) gene have been described (2114 variants, based on the CFTR1 database http://www.genet.sickkids.on.ca/, last accessed on July 2023). Their combined inheritance gives rise to genotypes with different courses and severities of symptoms in various affected organs (https://cftr2.org/) [1, 2, 4–8].

CFTR is a transmembrane protein channel that is expressed in most epithelial tissues, regulating ion exchange [9]. In particular, it is responsible for chloride, bicarbonate, and water efflux in the epithelia, preventing sodium-channel-driven water reabsorption, thus balancing water exchange and preserving tissue hydration and mucus fluidity and function. CFTR role is particularly relevant in respiratory, digestive and reproductive tracts, where mutated CFTR results in diseased tissues, with the most severe and life-threatening pathogenic effects occurring in the respiratory system. In fact, in the lungs of CF patients, the thick and dense mucus leads to infections, chronic inflammation, severe tissue damage, and finally, respiratory failure [1, 2].

The most frequent pathogenic CF genotypes have been investigated and are well characterized, with effective treatments consequently made available for the corresponding patients [10]. Therapies that are clinically approved to rescue the CFTR function comprise modulators that are used as single agents or in combinations [11]. The potentiator Ivacaftor/VX770 has been used as a single drug (Kalydeko™) or associated with correctors Lumacaftor/VX608 (Orkambi™) or Tezacaftor/VX661 (Symdeko™) and resulted in acceptable efficacy in specific genotypes for which the drug has indications. The triple combination Trikafta™, consisting of the two Tezacaftor and Elexacaftor/VX445 correctors in addition to the Ivacaftor potentiator, was approved in 2019 by FDA and in 2020 by the European Medicines Agency (EMA)with the name Kaftrio. This drug combination proved effective in specific patient categories, resulting in unprecedented experimental and clinical success and remarkable benefit for patients’ quality of life [12–15]. Currently, Trikafta™ is authorized for the treatment of homozygous F508del/F508del patients and all compound heterozygous patients carrying F508del and any other mutation on the second allele (F508del/any), representing 70–90% of the CF population according to specific geographic distribution.

On the contrary, rare variant patients remain excluded from approved modulator treatments to date [16, 17]. Most rare mutations still have unknown impact in terms of disease symptoms and severity and underlying molecular/cellular defects (https://www.cftr2.org). Nevertheless, due to the rarity of the specific genetic conditions, no evidence could be produced by clinical trials to support the use of Trikafta™ in the majority of rare genotypes lacking the F508del allele.

Preclinical experimentation could contribute to an increase in the functional characterization of CFTR variants and obtain information on their pharmacologic responses relative to currently approved or innovative compounds [18–21]. In this context, most in vitro systems for drug testing used so far are either inefficiently obtained (primary bronchial cells) or not based on patient cells (CFTR variant overexpression on commercial cell lines) [22]. Thus, more efficient and patient-specific in vitro approaches are required for the research of rare variants, considering the limited number of these patients and the paucity of their samples [21].

We, and others, have implemented an innovative approach in recent years, based on the conditional reprogramming of respiratory cells, to expand pulmonary and nasal airway stem cells in vitro [23–25]*.* This approach generates airway epithelial stem cells from the nasal epithelia of CF patients, exhibited an unprecedented efficiency with respect to the culture establishment (100%) and remarkable cell yields (> 5 × 10^8^ cell range starting from approximately 5 × 10^4^ cells). These cells have proved to be capable of differentiation under appropriate conditions, generating patient-like tissues and reproducing disease models with patient-specific properties, genotypes, and pathogenic defects [24]. These models, being obtainable virtually from each patient, were highly valuable for the assessment of drug response (theratyping) in both frequent and rare genotypes, with great implication for personalized CF therapy [24].

The L1077P (HGVS name: p.Leu1077Pro) CFTR pathogenic variant was first identified in 1994 as a transition T to C detected at nucleotide position 3362 in exon 17b of CFTR (HGVS name: c.3230T > C, exon 20). The transition causes a change from leucine (CTG) to proline (CCG) at position 1077 of the protein [26]. This CFTR variant is relatively frequent in CF patients from specific areas, including Italy [26–29]. It was first detected in a pancreatic sufficient CF patient with F508del on the other allele [26]. In a second study, L1077P mutants were described to cause severe disease when combined with another CF-causing mutant [30]. In our case series, it was present in 11 CF patients with pancreatic insufficiency (1 L1077P homozygote and 10 compound heterozygotes) [28]. In the CFTR2 database (https://cftr2.org/, last accessed July 2023), L1077P is reported as a CF-causing variant (85% of patients with pancreatic insufficiency) based on information about 93 CF patients worldwide. Further investigation is needed to better elucidate its clinical aspects, pathogenic molecular defects, and therapeutic response. The little information available, obtained in overexpression studies of the mutant L1077P variant in cell lines, shows the possibility of rescuing mutated CFTR membrane localization and function using experimental correctors that mitigate the interactions with proteostasis-involved proteins, preventing protein retention and degradation by proteasomes [30]. However, drug-induced CFTR rescue was not remarkable in these studies. Thus, further effort is still needed to obtain data to foster biochemical and functional characterization of this mutation, identify innovative and more effective therapeutics, or determine the therapeutic response to drugs that are already approved for other variants.

Here, we characterized the L1077P pathogenic variant in highly suitable personalized experimental disease models based on CF patients' nasal epithelia-derived airway stem cells obtained using the conditionally reprogrammed cell (CRC) approach, which was previously optimized [23, 24]. By using CRC-derived organoids and Air Liquid Interface (ALI) models, we focused on L1077P/L1077P homozygous, L1077P/W1282X and L1077P/R1066C compound heterozygous genotypes, characterizing the CFTR biochemical defects and their residual and pharmacologically rescued function in response to clinically approved CFTR modulators and their combinations, including the triple combination of Trikafta™-comprising drugs: Elexacaftor, Tezacaftor and Ivacaftor (ETI). A comparison with W1282X/W1282W and F508del/F508del homozygous genotypes was also performed. ALI-culture-based CFTR immunoblots and short-circuit current recordings in Ussing Chamber, as well as the Forskolin-induced swelling (FIS) of nasal organoids allowed the obtainment of results that support the implementation of Trikafta™-based personalized therapy for this orphan variant. The innovative methodology of CRC-based CF models proved to be powerful in vitro surrogates for patient clinical trials, and they can be considered as “in vitro clinical trials” or “patient-in-a-dish trials”, which are highly suitable approaches that can guide personalized CF therapies.

## Materials and methods

### Nasal brushing processing and CRC culture

Human nasal epithelial samples were provided in accordance with the consent procedures approved by the Internal Review Board of Policlinico Umberto I Hospital, Sapienza University of Rome (Ethics committee ref. 5660 prot 983/19 December 18th 2019 and ref. 6841 prot. 98/2023 February 8th 2023). Nasal epithelial cells were obtained via the cytology brushing (Doctor Brush, AIESI) of inferior turbinates from both nostrils; they were pooled into a single 15 ml conical tube filled with DMEM/F12 (Gibco, code 1320033) and 5 × antibiotics (Penicillin/Streptomycin and Amphotericin B). Samples were repeatedly washed, and the recovered cells were cultured using the Conditionally Reprogrammed Cell (CRC) methodology according to our previous protocols [23, 24]. Briefly, epithelial cells were co‐cultivated with irradiated (30 Gy) murine J2 Swiss 3T3 fibroblasts (Kerafast, Boston, MA, USA, code EF3003) in F medium (3:1 v/v F‐12 Nutrient Mixture Ham (Gibco, code 11765054): DMEM (Gibco, code 31765-027) supplemented with 5% Fetal Bovine Serum (Euroclone, South America, code ECS01180L); 0.4 μg/ml of hydrocortisone (Sigma, code H0888); 5 μg/ml of insulin (Sigma, code 91077C); 24 μg/ml of adenine (Sigma, code A2785); 8.4 ng/ml of cholera toxin (Sigma, code C8052) in the presence of 10 μM Rock inhibitor Y‐27632 (Selleck, Munich, Germany, code S1049); and 10 ng/ml of EGF (Peprotech, code AF100-15). Fibroblasts were cultured in DMEM supplemented with 10% characterized MSC-qualified USDA-approved Fetal Bovine Serum (HyClone™,Gibco, code 12662029) and irradiated when reached 80% confluence. All cells were maintained at 37 °C in a humidified incubator with 5% CO_2_.

### Differentiation of CRC in air–liquid interface (ALI) culture conditions

To induce differentiation, 1.1 × 10^5^ cells were plated in transwell inserts (Corning, code 3460) and cultured in CRC complete medium in both basal and apical chambers until confluence was reached (5–7 days); afterwards, the medium was replaced with PneumaCult-ALI Medium (Stem Cell Technologies, Cambridge, UK 05001) in the basal chamber, leaving the apical chamber empty for 4–6 weeks with medium replacement every other day. These cultures were used for immunoblotting assays. Alternatively, for their subsequent use in Ussing Chamber assays, smaller-size transwell Corning inserts (Corning, 3470) were used, and 5 × 10^4^ cells were plated for ALI-culture differentiation.

### CFTR mutational analysis

Genomic DNA was extracted from the CF-CRC cells using the QIAamp DNA Blood midi kit (Qiagen, Hilden, Germany 51183), and fluorimetric quantification was performed (Qubit, Invitrogen, CA, USA). Proximal 5’-flanking, all exons and adjacent intronic zones, and the 3′-UTR of the CFTR gene (RefSeq NM_000492.4, NG_016465.4) were sequenced using the Sanger cycle sequencing protocol (ThermoFisher Scientific, Waltham, MA, USA), as previously described [31–33] and a genetic analyzer (ABI PRISM 3130*xl*; Applied Biosystems, Foster City, CA, USA). Genotype analysis was completed using multiplex ligation-dependent probe amplification (SALSA MLPA probemix P091 CFTR, MRC Holland, Amsterdam, the Netherlands, EKI-FAM, P091-100).

### CFTR expression analysis

RNA was extracted from CF-CRC cells using the RNeasy mini kit (Qiagen, Hilden, Germany, 74104). It was reverse transcribed by the iScript cDNA Synthesis kit (Bio-Rad, Hercules, CA, USA, 170–8891). Retrotranscription was performed using 1 μg of total RNA in 5.5 μl, 4 μl of 5 × iScript reaction mix, 1 U of iScript reverse transcriptase in 1 μl, and 9.5 μl of H_2_O in a final volume of 20 μl, according to the manufacturer’s instructions. The reactions were incubated in a PTC 100 thermocycler (Bio-Rad) according to the following program: 5′ 25 °C, 30′ 42 °C, and 5′ 85 °C.

For qualitative CFTR expression analyses, the amplification of the CFTR cDNA using an RT-PCR procedure was performed according to our protocol [34]*.* Briefly, a PCR mix was prepared in a final volume of 15 µl containing the following: 2.5 µl of cDNA mix, 175 µM of each dNTP (Fermentas, Waltham, MA, USA, FL-50R0181), 1.5 mM of MgCl_2_, 6 pmol of each primer, and 0.5 U GoTaq hot start polymerase with 1× manufacturer’s buffer (Promega, Madison, Wisconsin, USA, M5006). A PTC100 thermocycler (Bio-Rad, Hercules, CA, USA) was used with the following PCR cycle: 2′ 95 °C; 35 cycles of 45″ 94 °C, 1′ 30″ 60 °C, 2′ 30″ 72 °C, followed by 7′ 72 °C. The amplicons were subsequently analyzed using electrophoresis on a 3% agarose gel. The cDNA amplicons of interest were extracted from agarose and individually sequenced as described above.

For quantitative CFTR expression analysis, droplet digital PCR (ddPCR) was performed starting from the cDNA mix described above, and a TaqMan gene expression assay (code 4331182, ID Hs00357011_m1; ThermoFisher Scientific, Waltham, MA, USA) with a dye-labeled TaqMan probe FAM, according to the manufacturer’s instructions. The aqueous final reaction volume was 20 μL using 1 μL of cDNA mix, 10 μl of 2 × ddPCR Supermix for probes (no dUTP) (Bio-Rad, Hercules, CA, USA, 1863024), 1 μl of specific TaqMan probe assay, and 8 μl of H_2_O. According to manufacturer instructions, 70 μl of droplet generation oil for probes was added. The water-in-oil droplet emulsion was prepared using a QX200 Droplet Generator (Bio-Rad). The amplification step was performed using 40 μl of emulsion in a C1000 thermal cycler (Bio-Rad) using the following protocol: 10′ 95 °C; 45 cycles of 30″ 94 °C, 1′ 60 °C, followed by 10′ 98 °C. The ddPCR reactions were analyzed using the QX200 Droplet Reader and QuantaSoft software version 1.7.4 (both from Bio-Rad).

### Immunoblot and analysis of pharmacologic rescue of mature CFTR protein

For immunoblotting studies, 20 μg of total lysate proteins from each sample were resolved on 3–8% polyacrylamide gel electrophoresis NuPAGE Tris–Acetate (Invitrogen, Carlsbad, CA, USA, EA03752BOX) and transferred to nitrocellulose membranes (GGE Healthcare Life Science, AmershamTM ProtranTM, 10600018). The following primary antibodies were used: mouse monoclonal CFTR-596 (CFTR Antibody Distribution Program Cystic Fibrosis Foundation, UNC-Chapel Hill, dilution 1:2500), mouse monoclonal DM1A alpha Tubulin (Novus Biologicals NB-100–690, dilution 1:1000), and mouse monoclonal β-actin (Sigma-Aldrich A5441, dilution 1:10,000). Peroxidase-conjugated secondary antibodies were purchased from Amersham™ (NA931V, NA934V, dilution 1:4000).

For the evaluation of the ability of drugs to rescue CFTR protein maturation, CF-CRC cells differentiated in ALI cultures for 4–6 weeks were exposed to the following drugs for 48 h: 5 µM of VX809/Lumacaftor (Selleck Chemicals S1565), 10 µM of VX661/Tezacaftor (Selleck Chemicals, S7059), and 3 µM of VX445/Elexacaftor (Selleck Chemicals, S7059) or their combinations before cell lysis and lysates were processed as described above. The quantification of immunoblot band intensity was performed using Image lab software (Chemidoc XRS + , Biorad). To quantify CFTR maturation, the relative amount of the CFTR band-C protein was normalized to actin or tubulin measured in the same protein sample, and these levels were used for subsequent calculations.

### CRC-derived organoid generation and forskolin-induced swelling assay

Cells were suspended at 30,000 cells/100 µl in growth-factor-reduced matrigel (Corning 354,230) using vigorous but careful pipetting to generate a single-cell suspension while avoiding the generation of air bubbles. This mixture was seeded in 100 µl aliquots into 24-well plates, creating a spherical “drop” of matrigel. The plates were incubated at 37 °C and 5% CO_2_ for 30 min to allow the setting of matrigel. CRC medium was added to the wells to cover the matrigel drop. After 5–7 days, cells were shifted in PneumaCult–ALI Medium until mature 3D structure was formed (typically after 21 days, with the presence of a lumen and a slightly thickened spheroid wall, suggesting a pseudostratified epithelium with motile cilia visible under high-magnification microscope), and the medium was replaced every other day. After 21 days, for the FIS assay, organoids were pre-treated with Lumacaftor, Tezacaftor and Elexacaftor or their combinations for 48 h, at the same doses as described above, based on our previous results and in line with therapeutically effective doses. Then, spheroid images were captured (10X magnification) at time 0 (48 h after corrector treatment), using a time-lapse imaging station (Olympus, Tokyo, Japan). Each organoid was monitored, and after 2 days of subsequent stimulation with the same correctors and 5 µM of Ivacaftor VX770 (Selleck Chemicals, code S1144) and 10 µM of Forskolin (Selleck Chemicals, code S2449), new images were taken (indicated as T1 in figures) to monitor and assess spheroid swelling. N = 10 spheroids in each experimental condition for a total of 3 experiments were analyzed. Images were analyzed by manually delineating the outer area of each spheroid using ImageJ software before and after the second treatment in order to avoid potential bias due to organoid size heterogeneity. Spheroid outer area data (basal and after stimulation) were imported into Microsoft Excel, and the fold change was calculated for each individual spheroid with respect to non-treated organoids.

### Short circuit currents recordings in Ussing Chamber assay

ALI-culture differentiated cells grown for at least 4 weeks in Corning 3470 insert transwells were left untreated (control) or pre-treated with the correctors Lumacaftor, Tezacaftor, Elexacaftor and the potentiator Ivacaftor for 2 days.Transwell membranes were mounted on a slider (P2302T; 0.33 cm^2^ aperture) in an Ussing chamber (P2300, PI). The transepithelial voltage was short-circuited with a voltage clamp (VCC MC8 Multichannel Voltage/Current Clamp, Physiologic Instruments). The offset between voltage electrodes and fluid resistance was cancelled before experiments. Recordings were performed filling both apical and basolateral hemi-chambers with 5 ml of a solution containing the following: 126 mM of NaCl, 0.38 mM of KH_2_PO_4_, 2.13 mM of K_2_HPO_4_, 1 mM of MgSO_4_, 1 mM of CaCl_2_, 24 mM of NaHCO_3_, and 10 mM of glucose, with a final pH of 7–7.3. Both sides were continuously bubbled with a gas mixture containing 5% CO_2_ and 95% 0_2_, and the temperature of the solution was maintained at 37 °C. Subsequently, the ENaC blocker Amiloride (100 µM), the cAMP agonist forskolin (FSK; 10 µM), and at the end of the test, the CFTR inhibitor CFTR 172inh 10 µM were added both to the apical and basolateral sides. The short-circuit current was acquired and analyzed using Acquire & Analyze software Version 2.3.8 (Physiologic Instruments). Raw tracings were processed using GraphPad Prism Version 8.0.0 for Windows as we previously reported [35]. We calculated the Isc changes (ΔIsc) by taking the difference in the Isc recorded after adding Forskolin or CFTR 172 inh.

## Results

### Patient genotypes, clinical status, and therapeutic regimens

Three CF patients (#1, #2 and #4) bearing the mutated L1077P CFTR-allele (legacy name) were the objects of this study. Patient 3, with genotype W1282X/W1282X, was selected and studied to be compared with the L1077P/W1282X genotype (Table 1).

**Table 1:** Patient clinical data

Patient	#1	#2	#3	#4
CFTR genotype (DNA level)	c.[3230T > C];[3230T > C]	c.[3230T > C];[3846G > A]	c.[3846G > A];[3846G > A]	c.[3230 T > C]; [3196C > T]
CFTR genotype (protein level)	p.[Leu1077Pro];[Leu1077Pro]	p.[Leu1077Pro];[Trp1282*]	p.[Trp1282*];[Trp1282*]	p.[Leu1077Pro];[Arg1066Cys]
Age (years)	49	42	42	22
Gender	M	M	M	M
Age at the first symptoms	1 month	2 months	2 months	1 month
Body mass index (BMI)	24.65	22.42	22	20.23
Sweat chloride (mEq/L)	87	120	77	97
Upper airways				
Chronic rhinosinusitis	Yes	Yes	Yes	Yes
Nasal polyposis	No	Yes	No	Yes
Lung				
Bacterial colonization	Mucoid Pseudomonas Aeruginosa	Mucoid PA	Mucoid PA	Mucoid PA
Bronchiectasis	Yes	Yes	Yes	Yes
Chronic pneumonia	Yes	Yes	Yes	Yes
Fibrosis	Yes	Yes	Yes	Yes
FEV1 (percent of predicted)	51	43	26	72
Other		Pulmonary hypertension	Pulmonary hypertension	
Pancreas/liver/stomach				
Chronic/recurrent pancreatitis	No	No	No	No
Pancreatic exocrine insufficiency	Yes	Yes (adipose degeneration)	Yes (adipose degeneration)	Yes (adipose degeneration)
Diabetes mellitus (CFRD)	Yes (insulin-dependent)	Yes (insulin-dependent)	Yes (insulin-dependent)	No
Other		chronic gastritis	Liver disease; chronic gastritis	Liver disease
Intestine				
Meconium ileus	No	No	No	No
Distal intestinal obstructive syndrome (DIOS)	No	No	No	Yes
Abdominal pain	No	No	Yes	Yes
Reproductive system				
Congenital Bilateral Absence of Vas Deferens (CBAVD)	Yes	Yes	Yes	Yes
Therapy	Pancreatic supplements; insulin; respiratory physiotherapy; mucoregulators (dornase); antibiotics (alternative cycles of tobramycin and colestin, 1 cycle/year I.V. and chronically inhaled); continuous O_2_ therapy	Pancreatic supplements; insulin; respiratory physiotherapy; mucoregulators (dornase); antibiotics (alternative cycles of tobramycin and colestin, 3 cycles/year I.V. and chronically inhaled); ursodeoxycholic acid; continuous O_2_ therapy	Pancreatic supplements; insulin; respiratory physiotherapy; mucoregulators (dornase); antibiotics (alternative cycles of tobramycin and colestin, 1 cycle/year I.V. and chronically inhaled); continuous O_2_ therapy	Pancreatic supplements; respiratory physiotherapy; mucoregulators (7% hypertonic saline); antibiotics (chronically inhaled tobramycin and colestin cycles); ursodeoxycholic acid; no O_2_ therapy

Clinical information of patients included in the study

Patient 1 carries the c.3230T > C (p.Leu1077Pro) missense mutation in both CFTR alleles, thus being homozygous for this genetic variant. This mutated CFTR gene codes for a CFTR pathogenic variant that displays a substitution of amino-acid leucine 1077 with a proline (genotype L1077P/1077P—legacy name-, c.[3230T > C];[3230T > C] p.[Leu1077Pro];[Leu1077Pro]—HGVS name).

Patient 2 displays the compound heterozygous genotype carrying the c.3230T > C missense mutation on the first allele (coding for the L1077P pathogenic variant, as in Patient 1) associated with the c.3846G > A (p.Trp1282*; legacy name W1282X) non-sense mutation variant of CFTR on the second allele. This mutated CFTR gene codes for a CFTR pathogenic variant that is truncated at the NBD2 region due to the presence of a premature termination codon (PTC) instead of tryptophan in amino-acid position 1282 (genotype L1077P/W1282X—legacy name -, c.[3230T > C];[3846G > A] p.[Leu1077Pro];[Trp1282*]—HGVS name).

Patient 3 was selected for his homozygous W1282X/W1282X genotype (HGVS name: c.[3846G > A];[3846G > A] p.[Trp1282*];[Trp1282*]), useful to compare Patient 1 and Patient 2 with a patient with both alleles coding for the truncated protein.

Patient 4 was an additional patient with a genotype containing the L1077P variant. He displays a compound heterozygous genotype carrying the c.3230T > C missense mutation on the first allele (coding for the L1077P pathogenic variant, as in Patient 1 and 2) associated with the [3196C > T] (p.Arg1066Cys; legacy name R1066C) missense pathogenic variant of CFTR on the second allele. This mutated CFTR gene codes for a CFTR protein variant, displaying a substitution of amino-acid arginine 1066 with cysteine (genotype L1077P/R1066C—legacy name -, c.[3230T > C];[3196C > T], p.[Leu1077Pro];[Arg1066Cys]—HGVS name).

The clinical parameters of each patient are described in Table 1 and are indicative of severe disease for all patients. Patient 4 displays a less severe clinical status with improved respiratory function compared to other patients (Table 1).

### Tezacaftor plus Elezacaftor correctors induced marked CFTR protein correction in CRC-derived ALI cultures with L1077P/L1077P and L1077P/W1282X genotypes

**Fig. 1:**
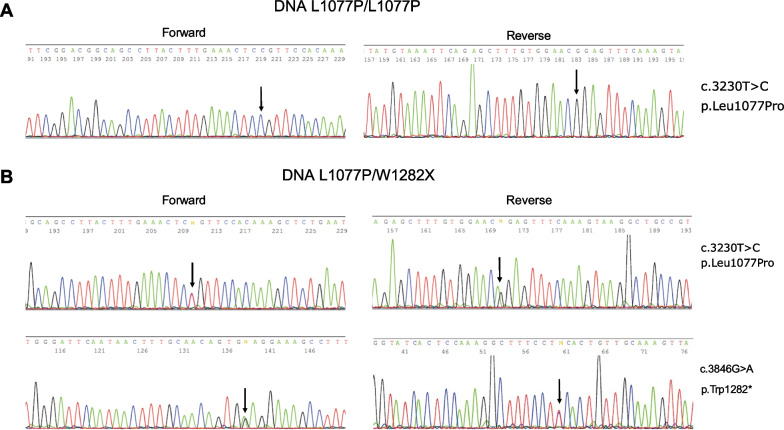
Confirmation of patients’ genotypes in CRC cells after in vitro expansion. In each panel, the sequencing results of the CFTR regions of interest (including the pathogenic variants), starting from the genomic DNA of CRC cells, are shown (forward sequencing in the left panels and reverse sequencing in the right panels). **A** CRC cells with homozygous genotype L1077P/L1077P. **B** CRC cells with compound heterozygous genotype L1077P/W1282X (the region of the L1077P pathogenic variant is shown in the upper panels; the region of the W1282X pathogenic variant is shown in the lower panels). The arrows indicate the mutated nucleotides; on the right side of the panels, the HGVS name of each pathogenic variant is shown in both nucleotidic and aminoacidic notation

Nasal brushing cells from patients with L1077P/L1077P and L1077P/W1282X genotypes (Patient 1 and Patient 2 in Table 1) were subjected to CRC culture conditions following our previously published protocols, determining the in vitro high expansion of respiratory basal stem cells [23, 24]. In vitro expanded cells were banked (10 vials of CRC cells were stored for each patient sample, yielding, as an approximate estimation, at least > 5 × 10^8^ CRC), following validation for stem cell and epithelial marker expression confirming their basal cell nature based on our previously studies and as reported in Additional file 1: Fig. S1 [24]. CFTR gene sequence was performed to detect and confirm pathogenic variants previously emerged during diagnostic procedures based on patient leucocytes, following our previously published protocols [24, 32, 33, 36]. Diagnostic pathogenic variants were confirmed in patient-derived cells after in vitro expansion, demonstrating that CRC cells maintain the patient genetic alterations after prolonged culture, in agreement with our previous data, thus constituting a suitable ex vivo disease model for the investigation of CFTR variants. Figure 1A shows the DNA sequence performed for the region of interest encompassing nucleotide c.3230 for Patient 1, and Fig. 1B shows genomic DNA sequences performed for the regions of interest encompassing mutated nucleotides c.3230 and c.3846 present on the two different mutated alleles in Patient 2, both starting from genomic DNA extracted from CRC cells. CFTR gene sequence analysis in CRC cells confirmed the patient’s diagnostic mutations: c.3230T > C substitution on both alleles of Patient 1; c.3230T > C and c.3846G > A substitutions in the two alleles of Patient 2.

**Fig. 2:**
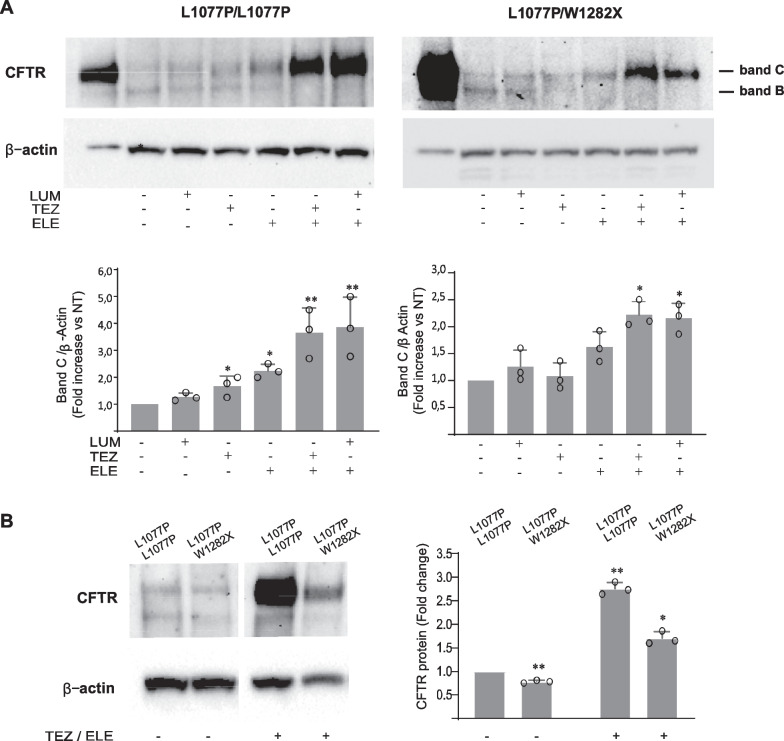
Evaluation of CFTR protein expression and maturation rescue induced by modulators. Immunoblot analyses of expression levels and molecular mass of CFTR protein in the different control or drug-treated ALI-differentiated cells are shown. **A** CFTR immunoblot images of L1077P/L1077P homozygous (Patient 1) and L1077P/W1282X compound heterozygous (Patient 2) genotypes as indicated, according to Table 1 (upper panels). Calu3 cells were loaded as CFTR molecular weight control (first lane of each immunoblot). The densitometric quantification is shown below the immunoblot. **B** Comparison of CFTR expression at protein level, by western blot (left) and densitometric evaluation (right) in ALI-differentiated cells with the two indicated genotypes, with or without Tezacaftor and Elexacaftor. The immunoblot image was cropped to remove samples that are unrelated to the experiment. For both A and B, β-Actin is shown for equal loading; the drug-treatments are indicated at the bottom; mean ± SD of three independent experiments (circles) is shown; Student’s t-test (with respect to untreated control): *p < 0.05; **p < 0.01. *LUM* Lumacaftor, *TEZ* Tezacaftor, *ELE* Elexacaftor; band C: mature CFTR; band B: immature CFTR. *NT* untreated control

The genotype of CRC cells was confirmed by sequencing also for the third W1282X/W1282X and fourth L1077P/R1066C patients (not shown). ALI cultures were established following our previous protocols [24]; validated for differentiation markers expression (Additional file 1: Fig. S1); used to characterize the L1077P CFTR protein defect and to determine its basal expression, maturation, and possible rescue by clinical correctors (used as single agents or in combination). Specifically, ALI-cultured cells (4 weeks differentiation) were treated for 2 days with the correctors Lumacaftor, Tezacaftor, or Elexacaftor as single agents, with the clinical combination of correctors Tezacaftor + Elexacaftor or with the experimental corrector combination Lumacaftor + Elexacaftor. Control or drug-treated cells were used to determine CFTR protein expression levels and molecular weight in immunoblot. In particular, the presence and intensity of immature (band B) and mature (band C) CFTR proteins were assessed and quantified in control and drug-treated cells. The increase in CFTR band C (normalized vs. housekeeping protein) was considered indicative of the extent of residual wild-type protein (in the control sample) or protein induction/correction (in drug-treated samples). Our results showed that, in both L1077P/L1077P and L1077P/W1282X genotypes, the basal expression of CFTR was very low, compatible with minimal function pathogenic variants and in line with the severe form of the disease affecting both patients (Fig. 2).

In L1077P/L1077P cells (Fig. 2A, left panels), treatment with drugs used as single agents did not result as significantly effective in modulating CFTR expression or induced a slight increase in band C in the case of ELE, while treatment with the Tezacaftor + Elexacaftor corrector combination determined a prominent induction of band C, indicating a strong ability of these correctors to induce the L1077P variant correction or decreased degradation. A similar marked efficacy in CFTR induction/correction was visible following the Lumacaftor + Elexacaftor-combined treatment.

In Patient 2-derived cells (L1077P/W1282X compound heterozygous genotype), the truncated protein, lacking 198 amino-acids in the C-terminal portion, was expected to appear with slightly lower molecular weights compared to full-length proteins in immunoblot both in its immature and mature forms. The immunoblot of untreated samples, however, revealed very faint bands corresponding to band C and B of the expected full-length protein encoded by the L1077P allele while truncated protein bands, possibly coded by the PTC mutated allele, were not visible at the expected molecular weights [37, 38]. Treatment with single correctors did not determine a significant increase in CFTR band C, while treatment with either corrector combinations (Tezacaftor + Elexacaftor and Lumacaftor + Elexacaftor) induced a marked enhancement of CFTR band C. In contrast to the increased amounts of full-length proteins deriving from the L1077P allele, the expected truncated W1282X protein was undetectable even after drug treatment (Fig. 2A right panels).

CFTR protein levels in untreated cells were generally low in the two genotypes, in line with a generally low basal expression of the mutated CFTR, and this is possibly due to the degradation of the abnormal CFTR protein. A direct comparison of CFTR protein levels revealed a reduced quantity in the L1077P/W1282X genotype with respect to the L1077P/L1077P genotype. (Fig. 2B). This effect was more visible in drug-treated cells, where the degradation process of abnormal proteins is expected to occur at lower extent. Indeed, immunoblot results showed that the corrector-induced CFTR band C levels were very high in the presence of two copies of L1077P, and the levels were lower in the compound heterozygote with a single L1077P allele (although markedly increased by treatment) (Fig. 2B).

**Fig. 3:**
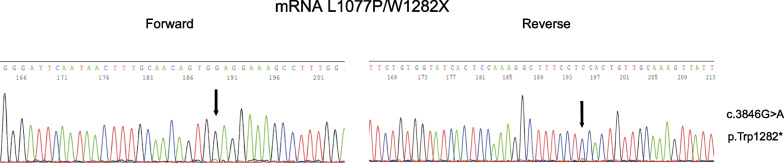
mRNA analysis of the W1282X region in the L1077P/W1282X compound heterozygous patient. In each panel, sequencing results of the amplicon of the CFTR region of interest (including the W1282X), obtained via RT-PCR starting from mRNA of CRC cells with L1077P/W1282X genotype, are shown (forward sequencing in the left panel; reverse sequencing in the right panel). The arrows indicate the position of the variant; on the right of panels, the HGVS name of the pathogenic variant is shown in both nucleotidic and aminoacidic notation

To determine whether the undetectable levels of the truncated W1282X protein depended on its degradation due to uncorrected folding or were consequent to reduced expression/degradation of PTC-mutated RNA, we performed mRNA analysis. As reported by others, mRNA expression from PTC CFTR variants may be very low in the case of W1282X due to the PTC-dependent activation of the Non sense-Mediated RNA-Decay (NMD) pathway [37, 39–41]. Indeed, we observed highly reduced levels of the W1282X CFTR mRNA, as visible in the electropherogram images of cDNA sequencing (Fig. 3). In fact, the expected nucleotide A peak at position 3846, corresponding to the nonsense mutation (G > A), is barely visible compared to the high peak of the G nucleotide deriving from the transcription of the L1077P allele, which is wild type at the same nucleotide position (Fig. 3). These data suggested that the undetectable levels of the truncated W1282X protein might depend on the reduced levels of the corresponding mRNA.

### Triple combination Elexacaftor + Tezacaftor + Ivacaftor (ETI) induced marked CFTR functional rescue in CRC-derived organoids with L1077P/L1077P and L1077P/W1282X genotypes in Forskolin-Induced Swelling assay

**Fig. 4:**
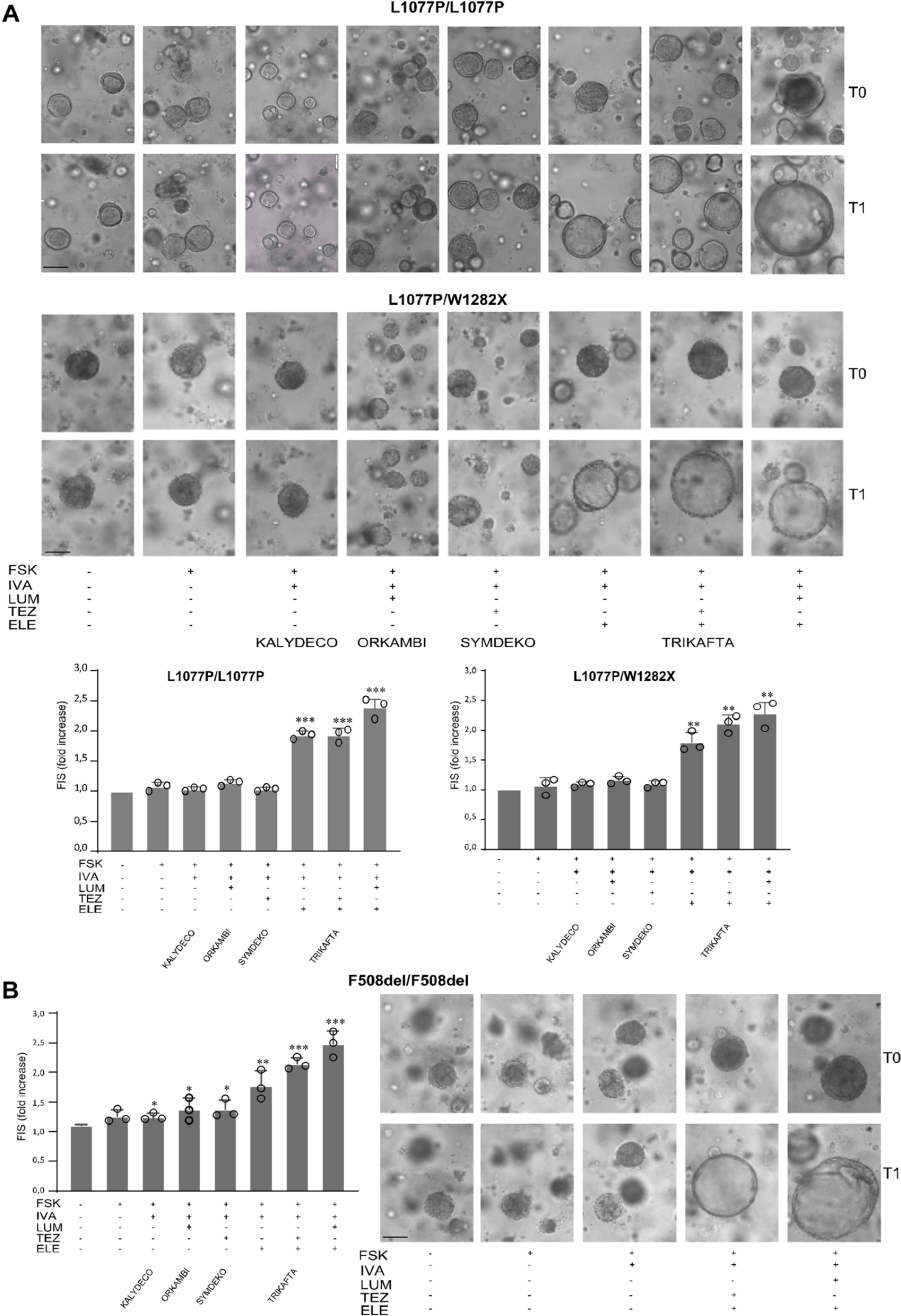
Evaluation of CFTR function recovery induced by modulators in organoids. **A** Forskolin-induced swelling (FIS) in CRC-derived organoids. Representative images of organoid FIS are reported relative to L1077P/L1077P homozygous (Patient1) and L1077P/W1282X compound heterozygous (Patient 2) genotypes, as indicated. The same organoids, monitored and measured after 48 h of exposure to the indicated correctors before (T0) and after 48 h of correctors/Forskolin/Ivacaftor (T1) treatments, are shown. In the bar diagrams, the quantitative responses of the organoid samples for the indicated drugs/drug combinations are shown. Mean ± SD of three independent experiments is shown (circles). Measures are relative to the increase in the organoid outer area (mean of at least ten organoids per condition) after stimulation with the indicated drugs. **B** FIS assay was performed for an F508del/F508del homozygote genotype (as reference) following the same procedures as in A. Student’s t-test (in respect to untreated control): *p < 0.05; **p < 0.01; ***p < 0.001. FSK: Forskolin; IVA: Ivacaftor; LUM: Lumacaftor; TEZ: Tezacaftor; ELE: Elexacaftor. Scale bars (100 µm) are indicated on the lower-left organoid image for each genotype and are representative of all organoid images

We next investigated whether the biochemical protein correction of CFTR observed in the immunoblot was associated with the rescue of CFTR function. CRC-derived organoids were treated with correctors, with the potentiator Ivacaftor and with the membrane-permeable cAMP activator Forskolin, to activate the CFTR channel opening. Organoid size was assessed before and after 48 h of stimulation with Forskolin/Ivacaftor to determine organoid swelling (FIS assay). The same organoids were traced and measured before and after Forskolin treatment in order to avoid biases due to the heterogeneity of the organoid population to obtain more reliable results. ETI induced a marked swelling of organoids, with the mean organoid size increasing about twofold after treatment in both genotypes (Fig. 4A). Similar results were also observed after treatment with the Elexacaftor–Lumacaftor–Ivacaftor combination, in line with biochemical results. The single corrector in combination with Ivacaftor did not substantially increase the organoid size, except for ELE, which showed higher activity that was, however, lower than the Tezacaftor + Elexacaftor or Lumacaftor + Elexacaftor corrector combinations. To compare the level of L1077P variant rescue to a benchmarked referenced functional rescue of a common mutant, we assessed modulator response in CRC-derived organoids with F508del/F508del genotype. As shown in Fig. 4B, the F508del homozygous genotype displayed a marked response to ETI in the FIS assay, with an extent comparable to L1077P-bearing genotypes, suggesting that the ETI response observed in L1077P-bearing genotypes may have potential clinical impact.

### Triple combination ETI induced marked CFTR functional rescue in CRC-derived ALI cultures with L1077P/L1077P and L1077P/W1282X genotypes in a short circuit current recording assay in the Ussing Chamber

In order to validate and confirm organoid FIS results, the ability of ETI and ELI to rescue CFTR function was also assessed in the short-circuit current recording assay of ALI cultures in the Ussing chamber.

ALI cultures derived from L1077P/L1077P, L1077P/W1282X and F508del/F508del (for comparison) patients were treated for 48 h with the selected compounds or the combinations ETI or ELI and then mounted in chambers for transepithelial short-circuit current recordings. During recordings, epithelia were first treated with the ENaC inhibitor Amiloride in order to allow the selective measure of the current passage specifically dependent on CFTR, stimulated with Forskolin, and finally blocked with the specific CFTR inhibitor—172 (inh172). The amplitudes of the current increase after Forskolin administration and the current drop subsequently induced by the CFTR inhibitor were taken as parameters reflecting CFTR function.

**Fig. 5:**
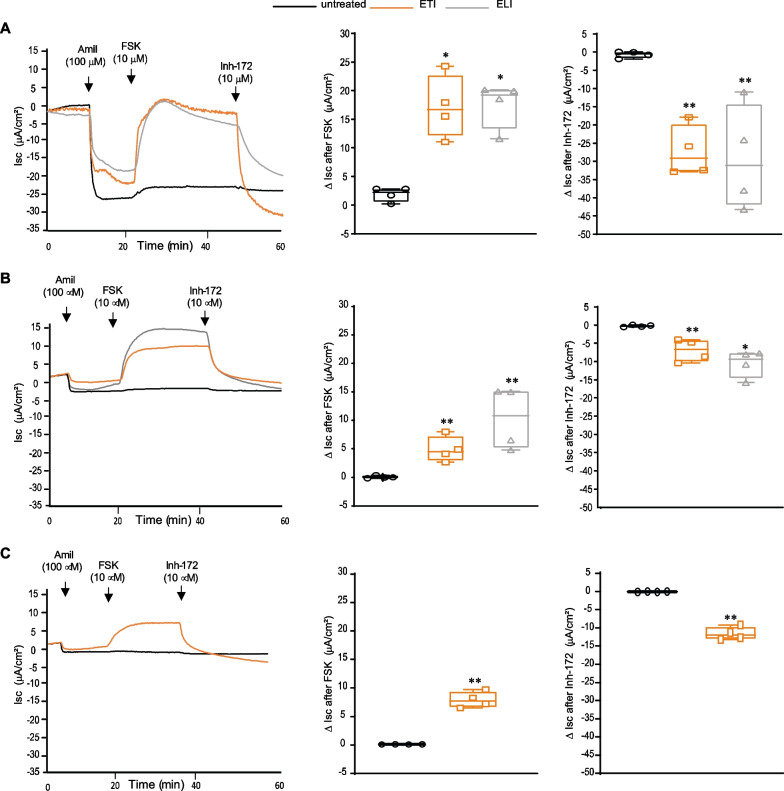
Evaluation of CFTR function recovery induced by modulators in Ussing Chamber recordings. Short-circuit current recording assay in the Ussing chamber showing the current curve modification after drug treatments for the L1077P/L1077P homozygous (**A** left curve), L1077P/W1282X compound heterozygous (**B** left curve) genotypes and for an F508del/F508del genotype (**C** left curve) as reference for clinically responder genotype. Black lines correspond to untreated samples, orange lines to ETI treatment, and grey lines to ELI treatment. Drug concentrations and times of drug additions (arrows) are indicated. Histograms represent the quantification of the positive variation of current passages after Forskolin (middle) and negative variation after Inh-172 (right) administration. Mean ± SD of four independent experiments is shown. Student’s t-test (with respect to the untreated control): *p < 0.05; **p < 0.01

In both genotypes bearing the L1077P allele (Fig. 5A, B), a prominent variation of current passage was induced after Forskolin administration specifically in ETI and ELI pre-treated ALI cultures compared to the untreated controls, while current passage variation was dramatically abolished by CFTR-specific inhibition with inh-172 in both genotypes. These findings indicate a specific and marked rescue of CFTR function by these drug combinations. In contrast, cells under control conditions showed nearly negligible response both to CFTR stimulation and inhibition. These results demonstrated a prominent, specific and modulator-dependent rescue of CFTR function in the L1077P CFTR variant either in double or single copy, in line with biochemical and organoid swelling results.

As a comparison, shown in Fig. 5C, ALI cultured cells with the F508del homozygous genotype displayed a marked response to ETI in the Ussing chamber assay, comparable to L1077P-bearing genotypes, suggesting that the ETI response observed in L1077P-bearing genotypes may have potential clinical impact.

### W1282X CFTR variant is unresponsive to ETI and showed reduced RNA and protein expression

In order to further clarify the possible contribution of the W1282X variant in the drug-induced rescue of CFTR function in the compound heterozygous genotype L1077P/W1282X, we generated a suitable CF model that carried the W1282X/W1282X homozygous genotype as an experimental control. Thus, we expanded CRC cells from the nasal brushing of a CF patient with W1282X/W1282X homozygous genotype and generated CRC-based ALI cultures and organoids to assess CFTR expression and modulator response in patient-derived cells. These models allowed the specific analysis of the W1282X variant expression, function, and response to ETI, being the only CFTR variant present, in double copy.

**Fig. 6:**
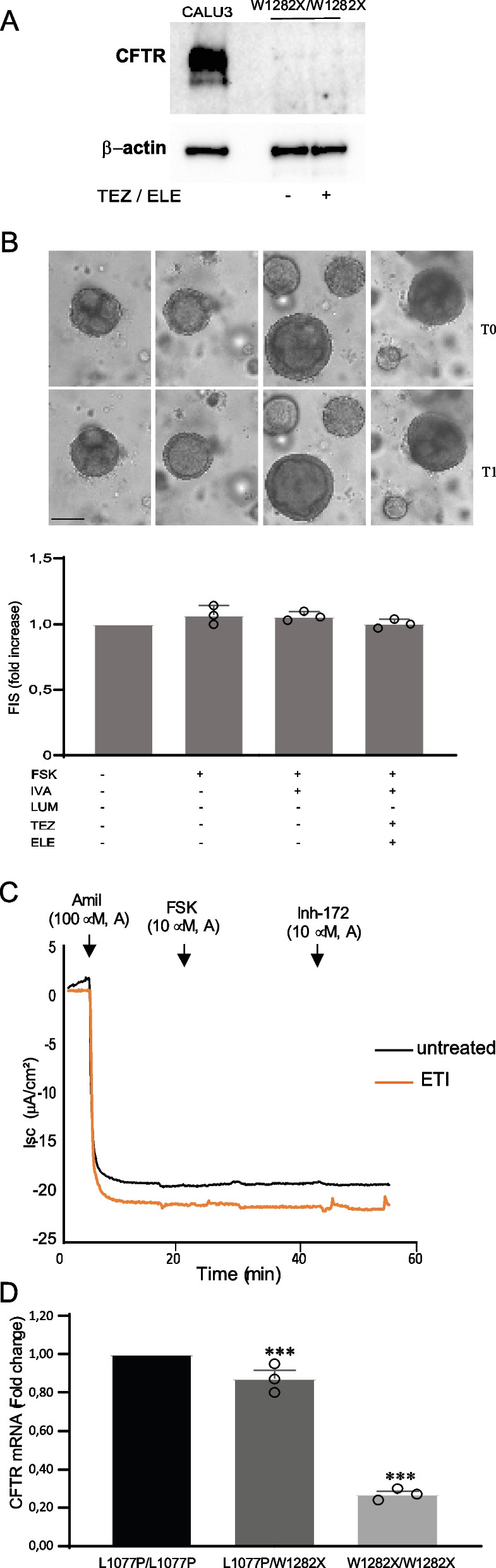
Evaluation of CFTR expression and ETI response in W1282X/W1282X homozygous genotype. **A** Evaluation of CFTR expression at the protein level, by Western blot of ALI-differentiated cells with the W1282X/W1282X genotype with or without Tezacaftor and Elexacaftor. Calu3 cells were loaded as a CFTR molecular weight control. β-Actin is shown for equal loading. **B** Evaluation of CFTR function recovery, by FIS assay, in (CF)- “culture reprogramming condition” (CRC)-derived organoids in the W1282X/W1282X genotype. The same organoids, monitored and measured before (T0) and after (T1) treatments are shown (upper images), as well as the increase in organoid area (lower histograms). Measures are relative to the increase in the outer organoid area (mean of at least 5 organoids per condition) after stimulation with the indicated drugs. Mean ± SD of three independent experiments is shown. **C** Evaluation of CFTR function recovery, in Ussing chamber recordings, in (CF) “culture reprogramming condition” (CRC)-derived cells, in W1282X/W1282X genotype; black line corresponds to untreated samples, orange line corresponds to the ETI treatment. **D** Comparison of CFTR expression at the RNA level by ddPCR in cystic fibrosis (CF) “culture reprogramming condition” (CRC)-derived air–liquid interface (ALI)-differentiated cells, with the three indicated genotypes. All quantitative differences were evaluated using Student’s t-test (with respect to the untreated control): ***p < 0.001. *FSK* Forskolin, *IVA* Ivacaftor, *LUM* Lumacaftor, *TEZ* Tezacaftor, *ELE* Elexacaftor, *TEZ/ELE* Tezacaftor + Tezacaftor; band C: mature CFTR; band B: immature CFTR. Scale bars (100 µm) are indicated on the lower-left organoid image and are representative of all organoid images

The W1282X CFTR protein was barely detectable in immunoblot at basal level in W1282X/ W1282X ALI-differentiated cells and did not display any increase after treatment with Elexacaftor + Tezacaftor correctors (Fig. 6A).

Moreover, ETI treatment was not effective in rescuing CFTR function in W1282X/W1282X organoids in the FIS assay, as the organoid size did not vary significantly in ETI-treated cells after Forskolin stimulation (Fig. 6B). Finally, Ussing chamber recordings confirmed the lack of short-circuit current passage variation either in control cells or in ETI pre-treated samples following stimulation with Forskolin (Fig. 6C).

We subsequently compared, in a quantitative manner, CFTR mRNA expression in ALI-cultured cells with the three different genotypes (L1077P/L1077P, L1077P/W1282X, and W1282X/W1282X) in order to verify whether CFTR expression levels and responses to drugs were strictly dependent on the presence and quantity of mRNA derived from L1077P allele(s), with a lack of contribution from the W1282X allele(s). CFTR mRNA expression in ALI-cultured cells, derived from the three patients with different genotypes, displayed a gradient of expression with high levels in L1077P/L1077P, decreased levels in L1077P/W1282X, and highly reduced levels in W1282X/W1282X samples, confirming that the CFTR protein in the compound heterozygote genotype was derived, essentially, from the transcription of the L1077P allele (Fig. 6D).

**Fig. 7:**
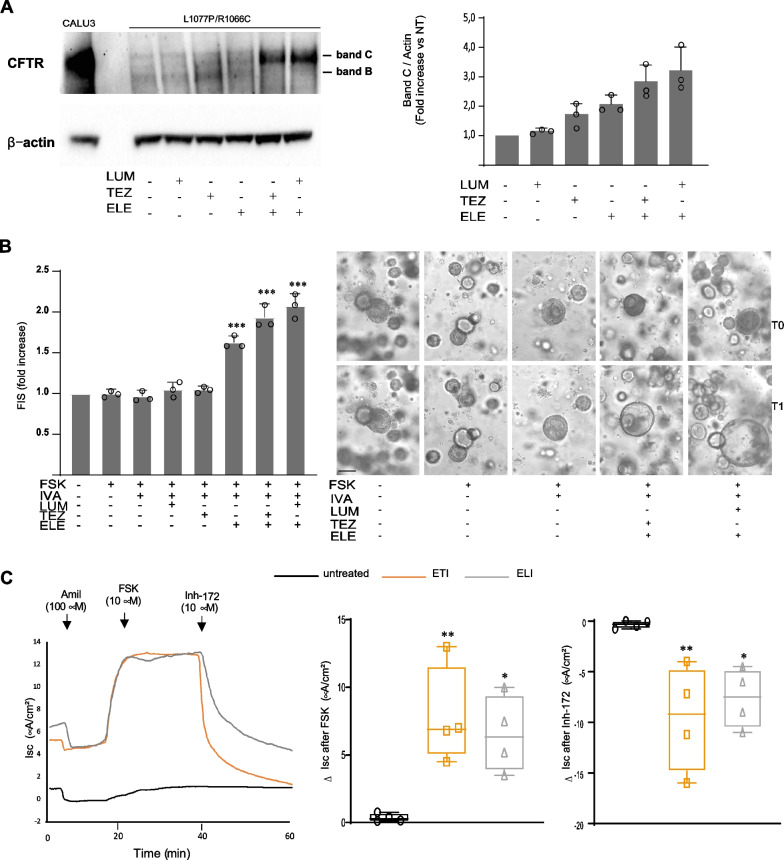
Evaluation of CFTR expression and ETI response in the L1077P/R1066C compound heterozygous genotype. **A** Evaluation of CFTR expression at the protein level by Western blot (on the left) and densitometric analysis (on the right). Mean ± SD and replicates (circles) are shown in ALI-differentiated cells with or without Tezacaftor, Lumacaftor and Elexacaftor. Calu3 cells were loaded as a CFTR molecular weight control. β-Actin is shown for equal loading. **B** Evaluation of CFTR function recovery, by FIS assay, in (CRC)-derived organoids. The same organoids monitored and measured before (T0) and after (T1) treatments are measured. Measures are relative to the increase in the organoid outer area (mean of at least ten organoids per condition) after stimulation with the indicated drugs (left diagrams). Mean ± SD of three independent experiments is shown (circles). Representative organoids of the more relevant conditions are shown (right panel). **C** Evaluation of CFTR function recovery in Ussing chamber recordings in CRC-derived ALI cultures; the black line corresponds to untreated samples, the orange line to the ETI treatment, and grey lines to the ELI treatment. All experiments were repeated four times. All quantitative differences were evaluated by Student’s t-test (with respect to untreated control): *p < 0.05; **p < 0.01; ***p < 0.001. *FSK* Forskolin; *IVA* Ivacaftor, *LUM* Lumacaftor, *TEZ* Tezacaftor, *ELE* Elexacaftor, TEZ/ELE: Tezacaftor + ; ETI: Elezacaftor + Tezacaftor + Ivacaftor; band C: mature CFTR; band B: immature CFTR. Scale bar (100 µm)

The intent of this study was to assess CFTR modulator response in a patient-personalized manner, considering also that L1077P is a rare mutation. Nevertheless, we asked if the ETI response would be reproduced in other individuals with the same CF-causing mutation background. Thus, we generated CRC-based models of ALI cultures and organoids and assessed CFTR expression and modulator response in a third genotype bearing L1077P allele, specifically the compound heterozygous L1077P/R1066C genotype, available at the CF center (Patient 4 in Table1). Our immunoblot performed in CRC-derived ALI cultures showed ETI ability to correct the CFTR protein conformation and to restore its expression (which was very low at basal conditions) with a marked increase in band C in ETI- and ELI-treated samples (Fig. 7A). Both ETI and ELI combinations demonstrated strong activity in rescuing CFTR function both in FIS (Fig. 7B) and Ussing chamber (Fig. 7C) assays, confirming the results obtained with L1077P/L1077P and L1077P/W1282X genotypes and suggesting that other genotypes bearing the L1077P allele may be responding to these drug combinations independently of the type of CFTR mutation on the second allele.

## Discussion

Trikafta™ is the third drug (after Symdeko™ and Orkambi™) approved by the FDA that rescues defects caused by the most frequent pathogenic variant: F508del. It is a triple combination of the correctors Elexacaftor and Tezacaftor in addition to the potentiator Ivacaftor and is superior to previous treatments. It improved respiratory function (FEV1), decreased exacerbations to a greater extent, and was highly effective also in CF patients who harbor only one copy of this mutation [42]. Therefore, the majority of CF patients have access to this therapy (representing 70–90% of the cystic fibrosis population according to specific geographic distribution). Nevertheless, the majority of genotypes lacking the F508del allele are ineligible for Trikafta™ or other treatments and currently remain orphans of modulator therapies. Among these rare genotypes, the L1077P CFTR pathogenic variant causes severe disease when combined with another CF-causing variant. Patients with the L1077P variant in their genotype undergo recurrent respiratory infections, chronic bronchial/lung inflammation and pancreatic insufficiency and intestinal problems, requiring oral supplements to help absorb the nutrients and vitamins, as well as oral pancreatic supplements to improve digestion.

Here, we used patient-specific in vitro disease models that we previously developed based on patient-derived nasal epithelial conditionally reprogrammed cells (CRCs): the 3D organoids and the ALI culture models [24, 24]. Here, we exploited these nasal CRC-derived in vitro models of CF for the characterization of the L1077P CFTR variant and the evaluation of its response to the ETI combination to obtain important inferences for future personalized patient therapy. Firstly, cells derived from two different patients carrying the homozygous L1077P/L1077P genotype and the compound heterozygous L1077P/W1282X genotype have been the object of this study, together with W1282X/W1282X and F508del/F508del genotypes used for comparison.

Biochemical and functional studies demonstrated a strong ability of ETI (and the experimental drug combination Lumacaftor + Elexacaftor + Ivacaftor) to rescue the CFTR L1077P variant in immunoblot, in the FIS assay of organoids, and in short-circuit recordings in Ussing chamber assay in both homozygous and compound heterozygous genotypes. These results, coupled with the observed dramatically reduced W1282X CFTR mRNA (compatible with NMD of PTC RNA, as described by others for this variant), showed that ETI induced a strong rescue of the L1077P variant even when present in single copy, displaying similar behavior with the F508del mutation. Thus, in line with what was already observed for F508del-bearing genotypes, our findings could open the way to the global approval of Trikafta not only for L1077P homozygous genotypes but also for genotypes that carry one copy of the responsive L1077P allele associated with any other variant on the second allele. This is also particularly relevant for genotypes carrying the other CFTR variant that is not correctable or not expressed, as in the case of nonsense mutations, expanding the cohort of patients that are eligible for modulator therapy. Thus, our studies may generate therapeutic opportunities for a reasonable number of patients that are currently not eligible for modulator therapy due to the assessment of a CFTR modulator response in a patient-personalized manner. Nevertheless, if the same theratyping results of ETI response obtained for these specific patients with the L1077P allele are reproduced in cells derived from different patients bearing the same allele, they might acquire increased reliability and be translated to other individuals with the same CF-causing mutation even though their cells are not specifically tested. For instance, patients that are too young or are unwilling or unable, for various reasons, to undergo nasal brushing and drug-testing experiments could benefit from the extrapolation of these results. Thus, we assessed CFTR expression and modulator response in a third genotypebearing L1077P allele, specifically the compound heterozygous L1077P/R1066C genotype (Patient 4 in Table 1). Our results showed the marked ability of ETI to rescue CFTR function in all CRC-based in vitro models, confirming the results obtained for other L1077P-bearing genotypes (Patient 1 and Patient 2) and suggesting that other genotypes that bear the L1077P allele may be responding to the triple drug combination independently of the type of CFTR mutation that is present on the second allele. Functional rescue by the triple combination of L1077P-containing genotypes was comparable to the F508del/F508del genotype rescue both in the FIS of organoids and Ussing chamber assay (Figs. 4B, 5C), further encouraging future Trikafta™ label expansion for the L1077P variant even if present in single-copy form, analogously with its indication for the F508del variant.

We also demonstrated in CRC-derived models with W1282X homozygous genotype, that this variant cannot be rescued by ETI, as a consequence of heavily compromised CFTR protein expression. In this genotype, undetectable levels of truncated protein in the immunoblot are likely to result from two combined mechanisms. The most significant quantitative effect seems to be due to the degradation of the PTC-mutated RNA (Figs. 3, 6D), with the consequence of a significantly reduced availability of mRNA for protein translation. Nevertheless, a reduced amount of PTC-mutated RNA seems to escape degradation, as revealed by a very sensitive ddPCR expression assay (Fig. 6D). However, this reduced quote of W1282X-mutated mRNA appears not to be properly translated/processed or the CFTR protein might be degraded as a consequence of its abnormal conformation in untreated cells. Consequently, the W1282X protein function cannot be rescued by correctors (Fig. 6A–C. These results are in line with the multiple mutational classes proposed for the W1282X variant [11]. Therefore, the basal and induced amount of CFTR protein present in L1077P/W1282X cells is likely to originate from the translation of the L1077P transcript. Additionally, the transcription of the L1077P allele in this compound heterozygote appears to be enhanced, possibly for the compensation of the compromised missense allele. In fact, by the ddPCR expression assay (Fig. 6D), only a limited reduction in CFTR mRNA is shown in the compound heterozygote with respect to the L1077P homozygote, in line with the limited reduction in protein (Fig. 2B). Importantly, these data indicate that the strong CFTR protein correction observed after treatment with Tezacaftor + Elexacaftor or Lumacaftor + Elexacaftor also in the compound heterozygous genotype is likely to depend on the activity of drugs on the L1077P protein produced by the single allele (Fig. 2).

These results highlight that a single L1077P allele may be sufficient to confer marked and satisfactory response to ETI in patient-derived CF cells. Therefore, the concept of an in vitro guided personalized therapy of non-F508del patients is strengthened and additionally extended to patients with a single targetable non-F508del allele. In addition, our results suggest that besides targeting two non-F508del alleles, the expression modulation of a single non-F508del allele also contributes to functional recovery. In this regard, an intriguing mechanism of single-allele expression enhancement, and consequently more effective therapeutic modulation, could be proposed. Due to its potential impact on the therapy of rare genotypes with only one non-F508del targetable allele, further studies appear mandatory.

Importantly, for the first time, CRC-derived ALI cultures were used in the Ussing chamber assay, extending its valuable predictive use for personalized medicine [43, 44]. Ussing chamber results confirmed the robust L1077P variant response to ETI, with great implications for therapeutic intervention.

Moreover, the obtainment of comparative results in the Ussing chamber assay and organoid FIS confers further reliability to the FIS assay of CRC-derived nasal organoids as a powerful and highly valuable ex vivo model of CF.

Organoids FIS assay proved to be suitable in providing the predictive indication of patient response to specific drugs, and these are helpful for guiding therapeutic choices for personalized patient therapy. Although all functional tests applied provided a similar indication of drug response, a standardization effort is needed to integrate their quantitative outcome in order to define the overall extent of in vitro response that need to be aligned with the parameters of clinical response.

Theratyping is routinely undertaken in CF care centers across the United States in order to guide patient therapy, while in Europe, CF patient response to modulators assessed in vitro cannot be formally exploited for clinical therapeutic indication. In fact, EMA and the Italian Medicines Agency (AIFA) regulation require patient enrollment in clinical trials for the subsequent label extension approval of modulators relative to specific genotypes. Based on the promising results of the patient-derived cell models described here, the hope is that the FIS assay performed in CRC-derived nasal organoids and assays based on ALI cultures may represent formally recognized in vitro tools for theratyping worldwide in the near future, with the acceptance of in vitro assay results as a valuable guide for the therapeutic management of rare patients.

Indeed, the high efficiency of the CRC-based model approach may satisfy several important needs. The first is that patient-derived models may be generated from any genotype in a personalized manner, allowing any in vitro drug testing in the cultures of a specific patient within his genetic background, known to modulate drug response in patients. Second, patient-specific cultures more faithfully reproduce the proper CFTR variant characteristics in terms of protein defects and expression. For instance, in some classes of mutations, such as nonsense mutations or splicing mutations, CFTR expression is also altered at the mRNA level (mRNA degradation due to NMD in the case of nonsense mutations, lack or very low amounts of wild-type mature mRNA expression in the case of abnormal splicing), affecting the levels of produced CFTR protein. In these contexts, CFTR variant overexpression in commercial cell lines or even in commercially available bronchial primary cells, which are often used, may be not optimal for theratyping and might provide incomplete or even biased results. Another important significant benefit of the CRC-based approach would be the ability to expand and preserve epithelial cells that retain their CF-causing genotype for future expansion and testing as new therapies develop.

Concerning the limits of theratyping approaches, it is worth mentioning that although several interconnected strategies are emerging for nasal cell expansion or for generation of nasal or rectal organoids, a common standardized approach of model generation and CFTR assay execution, shared by all laboratories, is still missing, calling for the need to develop common Standard Operating Procedures (SOPs) [45–47]. In this context, the unification into standard procedures, collecting the strengths of each methodology, might promote the prompt acceptance of in vitro theratyping as a powerful clinical therapeutic tool.

Moreover, the relevance of CFTR and other chloride channels that are expressed in organoid models has been reported and shed further light on their relevance in FIS assays [45]. Organoid generation has been further optimized to achieve higher CFTR expression and a more homogeneous degree of organoid differentiation [45]. Further investigation will be necessary to clarify the specific contribution of CFTR and other chloride channels in FIS assays in the models used in this study, although the use of modulators specific for CFTR should bypass this limit.

Another possible limitation of this studies, based on patient-derived models is that they imply the evaluation of the drug response of specific genotypes while not discriminating the contribution of the single pathogenic variants to genotype response.

However, as a point of strength, the theratyping of properly selected genotypes allowed the extrapolation of the contribution of each allele present in the studied genotypes to the modulator response. Of note, the use of patient-derived models is of higher value with respect to the heterotopic expression of variant CFTR cDNA, as in these models the endogenous expression of CFTR is not modified as by the overexpression and remains under the patient genetic background. Additionally, these models more faithfully mimic the patient tissue.

Finally, the comparison of drug response among uncharacterized and control genotypes with known clinical response (i.e., F508del) may provide information on the therapeutic relevance of preclinical findings. What would be useful is a direct comparison of these in vitro data with the clinical response to Kaftrio of the corresponding patients. Hopefully, this study will contribute to hastening the indication extension of Kaftrio by European and Italian regulatory agencies relative to L1077P-bearing patients, and this will in turn allow retrospective studies to assess the correspondence between in vitro drug testing and clinical response.

As a final conclusion, CRC-based models may be valuable tools that can guide personalized therapy, representing a non-invasive, rapid, suitable and valuable surrogate for clinical trials with great benefit for those rare patients who are still excluded from approved modulator therapies and do not have access to clinical studies.

### Supplementary Information

**Additional file 1: Figure S1. **Cystic fibrosis (CF) -“culture reprogramming condition” (CRC) characterization and generation of ALI-culture differentiated respiratory cell models. Results from one representative preparation (L1077P/L1077P genotype according to Table 1) are reported. A) Flow cytometry analysis of the basal cell markers nerve growth factor receptor (NGFR) and integrin α-6 (ITGA6) in CF-CRC and 4 weeks differentiated cells in air–liquid interface (ALI) culture condition. B) Immunofluorescence and confocal analysis for differentiation markers acetylated α-tubulin (green) and mucin5B (red) after ALI-culture differentiation. Magnification is 60× and orthogonal projection (Z) shows tissue thickness with nuclei (stained in blue with 4′,6-diamidino-2-phenylindole) at the bottom and differentiation antigens at the apical side. C) Immunoblot showing the loss of basal stem cell marker cytokeratin 5 (CK-5) and the acquisition of the differentiation markers of mature ciliated cells FOXJ and acetylated α-tubulin, after ALI differentiation; β-actin is shown for equal loading.

## Data Availability

Not applicable.

## References

[1] Elborn JS (2016). Cystic fibrosis. Lancet.

[2] Scotet V, L'Hostis C, Ferec C (2020). The changing epidemiology of cystic fibrosis: incidence, survival and impact of the CFTR gene discovery. Genes (Basel).

[3] McBennett KA, Davis PB (2022). Toward a broader understanding of cystic fibrosis epidemiology and its impact on clinical manifestations. Clin Chest Med.

[4] Sosnay PR (2013). Defining the disease liability of variants in the cystic fibrosis transmembrane conductance regulator gene. Nat Genet.

[5] Marson FAL, Bertuzzo CS, Ribeiro JD (2016). Classification of CFTR mutation classes. Lancet Respir Med.

[6] Laselva O (2022). Small-molecule drugs for cystic fibrosis: where are we now?. Pulm Pharmacol Ther.

[7] Britto CJ, Taylor-Cousar JL (2022). Cystic fibrosis in the era of highly effective CFTR modulators. Clin Chest Med.

[8] Joynt AT, Cutting GR, Sharma N (2022). Genetics of cystic fibrosis: clinical implications. Clin Chest Med.

[9] Amaral MD (2020). CFTR processing, trafficking and interactions. J Cyst Fibros.

[10] Corvol H (2016). Translating the genetics of cystic fibrosis to personalized medicine. Transl Res.

[11] Veit G (2016). From CFTR biology toward combinatorial pharmacotherapy: expanded classification of cystic fibrosis mutations. Mol Biol Cell.

[12] Middleton PG (2019). Elexacaftor–Tezacaftor–Ivacaftor for cystic fibrosis with a single Phe508del Allele. N Engl J Med.

[13] Keating D (2018). VX-445-Tezacaftor-Ivacaftor in patients with cystic fibrosis and one or two Phe508del alleles. N Engl J Med.

[14] Heijerman HGM (2019). Efficacy and safety of the elexacaftor plus tezacaftor plus ivacaftor combination regimen in people with cystic fibrosis homozygous for the F508del mutation: a double-blind, randomised, phase 3 trial. Lancet.

[15] Veit G (2020). Allosteric folding correction of F508del and rare CFTR mutants by elexacaftor–tezacaftor–ivacaftor (Trikafta) combination. JCI Insight.

[16] De Boeck K (2020). Cystic fibrosis in the year 2020: a disease with a new face. Acta Paediatr.

[17] Harutyunyan M (2018). Personalized medicine in CF: from modulator development to therapy for cystic fibrosis patients with rare CFTR mutations. Am J Physiol-Lung Cell Mol Physiol.

[18] Terlizzi V (2017). Genotype-phenotype correlation and functional studies in patients with cystic fibrosis bearing CFTR complex alleles. J Med Genet.

[19] Ramalho AS (2022). Assays of CFTR function in vitro, ex vivo and in vivo. Int J Mol Sci.

[20] Clancy JP (2019). CFTR modulator theratyping: current status, gaps and future directions. J Cyst Fibros.

[21] Amaral MD (2022). Precision medicine for rare diseases: the times they are A-Changin'. Curr Opin Pharmacol.

[22] Ramalho AS, Amato F, Gentzsch M (2023). Patient-derived cell models for personalized medicine approaches in cystic fibrosis. J Cyst Fibros.

[23] Sette G et al. Conditionally reprogrammed cells (CRC) methodology does not allow the in vitro expansion of patient-derived primary and metastatic lung cancer cells. Int J Cancer. 2018. 10.1002/ijc.31260 29341112

[24] Sette G et al. Theratyping cystic fibrosis in vitro in ALI-culture and organoid models generated from patient-derived nasal epithelial conditionally reprogrammed stem cells. Eur Respir J. 2021. 10.1183/13993003.00908-2021 PMC8675295 34413153

[25] Butler CR (2016). Rapid expansion of human epithelial stem cells suitable for airway tissue engineering. Am J Respir Crit Care Med.

[26] Bozon D (1994). Identification of four new mutations in the cystic fibrosis transmembrane conductance regulator gene: I148T, L1077P, Y1092X, 2183AA–>G. Hum Mutat.

[27] Castaldo G (2005). Comprehensive cystic fibrosis mutation epidemiology and haplotype characterization in a southern Italian population. Ann Hum Genet.

[28] Lucarelli M (2015). A genotypic-oriented view of CFTR genetics highlights specific mutational patterns underlying clinical macrocategories of cystic fibrosis. Mol Med.

[29] Lucarelli M (2017). A new targeted CFTR mutation panel based on next-generation sequencing technology. J Mol Diagn.

[30] Lopes-Pacheco M (2017). Combination of correctors rescues CFTR transmembrane-domain mutants by mitigating their interactions with proteostasis. Cell Physiol Biochem.

[31] Lucarelli M et al. Simultaneous cycle sequencing assessment of (TG)m and Tn tract length in CFTR gene. Biotechniques. 2002; 32(3): 540–2, 544–7. 10.2144/02323st06 11911657

[32] Lucarelli M (2006). A 96-well formatted method for exon and exon/intron boundary full sequencing of the CFTR gene. Anal Biochem.

[33] Ferraguti G (2011). A template for mutational data analysis of the CFTR gene. Clin Chem Lab Med.

[34] Auriche C (2010). CFTR expression and activity from the human CFTR locus in BAC vectors, with regulatory regions, isolated by a single-step procedure. Gene Ther.

[35] Comegna M (2021). Elexacaftor–Tezacaftor–Ivacaftor therapy for cystic fibrosis patients with the F508del/unknown genotype. Antibiotics (Basel).

[36] Lucarelli M (2016). the impact on genetic testing of mutational patterns of CFTR gene in different clinical macrocategories of cystic fibrosis. J Mol Diagn.

[37] Kim YJ (2022). Gene-specific nonsense-mediated mRNA decay targeting for cystic fibrosis therapy. Nat Commun.

[38] Sanderlin EJ (2022). CFTR mRNAs with nonsense codons are degraded by the SMG6-mediated endonucleolytic decay pathway. Nat Commun.

[39] Aksit MA (2019). Decreased mRNA and protein stability of W1282X limits response to modulator therapy. J Cyst Fibros.

[40] Venturini A (2021). Comprehensive analysis of combinatorial pharmacological treatments to correct nonsense mutations in the CFTR gene. Int J Mol Sci.

[41] Laselva O (2020). Functional rescue of c.3846G>A (W1282X) in patient-derived nasal cultures achieved by inhibition of nonsense mediated decay and protein modulators with complementary mechanisms of action. J Cyst Fibros.

[42] Bear CE (2020). A therapy for most with cystic fibrosis. Cell.

[43] Terlizzi V (2021). Effectiveness of elexacaftor/tezacaftor/ivacaftor therapy in three subjects with the cystic fibrosis genotype Phe508del/unknown and advanced lung disease. Genes (Basel).

[44] Terlizzi V (2021). Ex vivo model predicted in vivo efficacy of CFTR modulator therapy in a child with rare genotype. Mol Genet Genom Med.

[45] Amatngalim GD et al. Measuring cystic fibrosis drug responses in organoids derived from 2D differentiated nasal epithelia. Life Sci Alliance. 2022; 5(12). 10.26508/lsa.202101320 PMC9351388 35922154

[46] Conti J, Sorio C, Melotti P (2022). Organoid technology and its role for theratyping applications in cystic fibrosis. Children (Basel).

[47] Sachs N (2019). Long-term expanding human airway organoids for disease modeling. EMBO J.

