# Correction to: L1077P CFTR pathogenic variant function rescue by Elexacaftor–Tezacaftor–Ivacaftor in cystic fbrosis patient-derived air–liquid interface (ALI) cultures and organoids: in vitro guided personalized therapy of non-F508del patients

**DOI:** 10.1186/s12931-023-02535-x

**Published:** 2023-09-23

**Authors:** Stefania Lo Cicero, Germana Castelli, Giovanna Blaconà, Sabina Maria Bruno, Giovanni Sette, Riccardo Pigliucci, Valeria Rachela Villella, Speranza Esposito, Immacolata Zollo, Francesca Spadaro, Ruggero De Maria, Mauro Biffoni, Giuseppe Cimino, Felice Amato, Marco Lucarelli, Adriana Eramo

**Affiliations:** 1 Department of Oncology and Molecular Medicine, Istituto Superiore di Sanità, Rome, Italy; 2 Department of Experimental Medicine, Sapienza University of Rome, Rome, Italy; 3 Department of Molecular Medicine and Medical Biotechnologies, University of Naples Federico II, Naples, Italy; 4 CEINGEBiotecnologie Avanzate S.c.a.r.l, Naples, Italy; 5 Confocal Microscopy Unit, Core Facilities, Istituto Superiore di Sanità, Rome, Italy; 6 Dipartimento di Medicina e Chirurgia Traslazionale, Università Cattolica del Sacro Cuore, Rome, Italy; 7 Fondazione Policlinico Universitario ‘A. Gemelli’-IRCCS, Rome, Italy; 8 Cystic Fibrosis Reference Center of Lazio Region, AOU Policlinico Umberto I, Rome, Italy; 9 Cenci Bolognetti Foundation, Pasteur Institute, Sapienza University of Rome, Rome, Italy

**Correction to:** Stefania et al. Respiratory Research (2023) 24:217

10.1186/s12931-023-02516-0

Following publication of the original article [1], the authors identified an error in the authors name.

The given name and family name of all authors name were erroneously transposed.

The incorrect author name is: Lo Cicero Stefania^1^^†^, Castelli Germana^1^^†^, Blaconà Giovanna^2^, Bruno Sabina Maria^2^, Sette Giovanni^1^, Pigliucci Riccardo^1^, Villella Valeria Rachela^3^^,^^4^, Esposito Speranza^3^^,^^4^, Zollo Immacolata^3^^,^^4^, Spadaro Francesca^5^, De Maria Ruggero^6^^,^^7^, Biffoni Mauro^1^, Cimino Giuseppe^8^, Amato Felice^3^^,^^4^, Lucarelli Marco^2^^,^^9^^†^ and Eramo Adriana^1^^*^^†^

The correct author name is: Stefania Lo Cicero^1^^†^, Germana Castelli^1^^†^, Giovanna Blaconà^2^, Sabina Maria Bruno^2^, Giovanni Sette^1^, Riccardo Pigliucci^1^, Valeria Rachela Villella^3^^,^^4^, Speranza Esposito^3^^,^^4^, Immacolata Zollo^3^^,^^4^, Francesca Spadaro^5^, Ruggero De Maria^6^^,^^7^, Mauro Biffoni^1^, Giuseppe Cimino^8^, Felice Amato^3^^,^^4^, Marco Lucarelli^2^^,^^9^ and Adriana Eramo^1^^*^

The author group has been updated above and the original article [1] has been corrected.
