# Supplementary material for: L1077P CFTR pathogenic variant function rescue by Elexacaftor–Tezacaftor–Ivacaftor in cystic fibrosis patient-derived air–liquid interface (ALI) cultures and organoids: in vitro guided personalized therapy of non-F508del patients
Source: Respir Res. 2023 Sep 6;24:217. doi: 10.1186/s12931-023-02516-0 (PMC10483775; doi:10.1186/s12931-023-02516-0)
Supplement: Supplementary file 1 — Additional file 1: Figure S1. Cystic fibrosis (CF) -“culture reprogramming condition” (CRC) characterization and generation of ALI-culture differentiated respiratory cell models. Results from one representative preparation (L1077P/L1077P genotype according to Table 1) are reported. A) Flow cytometry analysis of the basal cell markers nerve growth factor receptor (NGFR) and integrin α-6 (ITGA6) in CF-CRC and 4 weeks differentiated cells in air–liquid interface (ALI) culture condition. B) Immunofluorescence and confocal analysis for differentiation markers acetylated α-tubulin (green) and mucin5B (red) after ALI-culture differentiation. Magnification is 60× and orthogonal projection (Z) shows tissue thickness with nuclei (stained in blue with 4′,6-diamidino-2-phenylindole) at the bottom and differentiation antigens at the apical side. C) Immunoblot showing the loss of basal stem cell marker cytokeratin 5 (CK-5) and the acquisition of the differentiation markers of mature ciliated cells FOXJ and acetylated α-tubulin, after ALI differentiation; β-actin is shown for equal loading. [file 12931_2023_2516_MOESM1_ESM.pdf]

Additional file 1 Materials

Antibodies used for immunoblot: rabbit polyclonal CK5 (Invitrogen PA5-32465, dilution 1:500), mouse monoclonal FOXJ1 (eBioscience Ab2A5, dilution 1:250), mouse monoclonal acetylated α-tubulin (Sigma T6793, dilution 1:1000) antibodies.

Additonal Methods

Flow cytometry.

For flow cytometry, 1x10^5 cells were incubated with the following antibodies: FITC-conjugated anti-mouse CD271/LNGFR (Miltenyi Biotech, dilution 1:10) or corresponding isotype control antibodies. Staining for integrin α6 was performed with rat monoclonal anti-integrin α6 Clone GoH3 (Abcam, dilution 1:100) and secondary goat anti rat Alexa Fluor 647 antibody (Invitrogen dilution 1:1000). Stained cells were analyzed with CytoFLEX flow cytometer (Beckman Coulter).

Immunofluorescence and Confocal Laser Scanning Microscopy (CLSM) analysis on ALI-cultures.

For immunofluorescence and CLSM analysis ALI-cultures were fixed with 2% paraformaldehyde for 30 min at room temperature and permeabilized with 0.5% Triton-X-100/ PBS for 10 min at room temperature. Primary mouse anti-acetylated α-tubulin (Clone 6-11B-1, Sigma T6793, dilution 1:50) and rabbit anti-Mucin 5B (Santa Cruz H300, cat. 20119, dilution 1:50). Antibodies were added in 0.5% Triton-X-100/3% BSA/3% FCS and incubated 90min at room temperature, followed by incubation with Alexa Fluor-488 F(ab)2 fragments of goat anti-mouse IgG and Alexa Fluor-594 F(ab)2 fragments of goat anti-rabbit IgG (Thermo Fisher Scientific), for 1h at room temperature. Nuclei were stained with 4',6'-diamidino-2phenykindole (DAPI) (Thermo Fisher Scientific). Membranes were finally cut from the scaffold, placed on glass slides and coverslips were mounted with Vectashield mounting medium for fluorescence (Vector Laboratories). CLSM observations were performed with a Zeiss LSM980 apparatus, using a 40x/1.40 NA oil objective and excitation spectral laser lines at 405, 488 and 594 nm. Image acquisition and processing were carried out using the Zeiss confocal software Zen 3.3 (Blue edition) and Adobe Photoshop CS5 software programs (Adobe Systems). Signals from different fluorescent probes were taken in sequential scan settings. Several cells for each labeling condition were analyzed and representative results are shown.

Legends to Additional Figures:

Legend to Figure S1

Cystic fibrosis (CF) -“culture reprogramming condition” (CRC) characterization and generation of ALI-culture differentiated respiratory cell models. Results from one representative preparation (L1077P/L1077P genotype according to Table 1) are reported. A) Flow cytometry analysis of the basal cell markers nerve growth factor receptor (NGFR) and integrin α-6 (ITGA6) in CF-CRC and 4 weeks differentiated cells in air–liquid interface (ALI) culture condition. B) Immunofluorescence and confocal analysis for differentiation

markers acetylated α-tubulin (green) and mucin5B (red) after ALI-culture differentiation. Magnification is 60× and orthogonal projection (Z) shows tissue thickness with nuclei (stained in blue with 4′,6-diamidino-2-phenylindole) at the bottom and differentiation antigens at the apical side. C) Immunoblot showing the loss of basal stem cell marker cytokeratin 5 (CK-5) and the acquisition of the differentiation markers of mature ciliated cells FOXJ and acetylated α-tubulin, after ALI differentiation; β-actin is shown for equal loading.

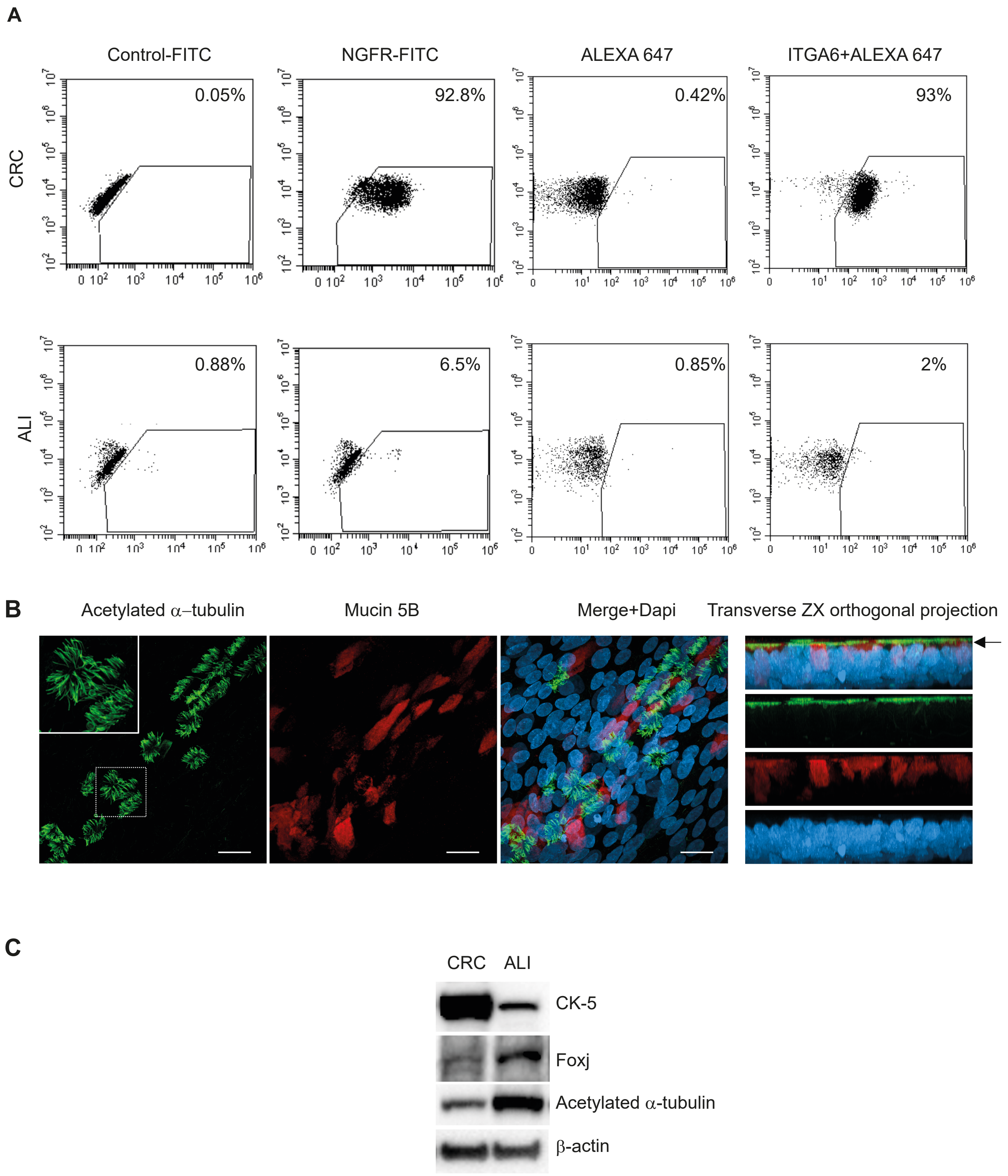
A
Control-FITC
NGFR-FITC
ALEXA 647
ITGA6+ALEXA 647
CRC
ALI
0.05%
92.8%
0.42%
93%
0.88%
6.5%
0.85%
2%
B
Acetylated α–tubulin
Mucin 5B
Merge+Dapi
Transverse ZX orthogonal projection
C
CRC
ALI
CK-5
Foxj
Acetylated α-tubulin
β-actin

Figure S1
